# Assessing non-dysfunctional attitudes toward sleep: psychometric properties of the Charlotte Attitudes Toward Sleep scale in Portuguese samples

**DOI:** 10.1186/s41155-024-00320-3

**Published:** 2024-09-14

**Authors:** Miguel Tecedeiro, Cátia Reis, João Marôco

**Affiliations:** 1 William James Center for Research, ISPA—Instituto Universitário de Ciências Psicológicas, , Sociais E da Vida, Avenida Jardim Do Tabaco 34, 1140-041 Lisbon, Portugal; 2 Católica Research Centre for Psychological, Family and Social Wellbeing, Universidade Católica Portuguesa, Palma de Cima, 1649-023 Lisbon, Portugal; 3 Instituto de Medicina Molecular João Lobo Antunes, Lisbon, Portugal; 4 Instituto de Saúde Ambiental, Faculdade de Medicina, Universidade de Lisboa, Lisboa, Portugal

**Keywords:** Sleep, Attitudes, CATS, KSQ, Validity, Reliability, Psychometry

## Abstract

**Objectives:**

To adapt the Charlotte Attitudes Toward Sleep (CATS) scale, the only self-assessment instrument measuring non-dysfunctional attitudes toward sleep, into Portuguese and to study its psychometric properties in a Portuguese sample.

**Method:**

A sample of 1858 participants, recruited through non-probabilistic methods, was randomly split in two subsamples; one was used to develop the CATS model, the other for testing model invariance. We used structural equation models to assess factorial validity, measurement invariance, and relationships with other variables (e.g., the Karolinska Sleep Questionnaire) through confirmatory factorial analysis and causal models using a robust maximum likelihood method with Satorra-Bentler correction.

**Results:**

The CATS factorial model showed excellent evidence of factorial validity (robust CFI = 0.987, TLI = 0.979, SRMR = 0.026, RMSEA = 0.043), good reliability indicators (*α* and *ώ*^1^ > 0.75), and strict invariance of measurement (|∆CFI|< 0.01). CATS factors were weak-to-moderate predictors of sleep behaviors (*β* < 0.4). The scale showed evidence of divergent validity with the Karolinska Sleep Questionnaire. Some items had significant sensitivity problems and/or did not have adequate factorial weights and had to be dropped from the model.

**Conclusions:**

The CATS is a new and promising scale with solid evidence of validity in terms of internal structure, but with sensitivity issues at item level. Further work should be carried out at item level to increase sensitivity and predictive validity, and further research with other samples, such as clinical sleep medicine patients, should be conducted.

**Supplementary Information:**

The online version contains supplementary material available at 10.1186/s41155-024-00320-3.

## Introduction

Sleep in humans is defined as a reversible, temporary, perceptual disengagement from the environment, accompanied by a reclining posture, inactivity, closed eyes, and a complex series of physiological and psychological processes (Sullivan et al., 2021). Humans spend about a third of their lives sleeping, and its importance in maintaining both physical and mental health has been well documented in both children and adults (WHO Technical Meeting on Sleep and Health, 2004).

Sleep is influenced by a multitude of variables, from genetics (Barclay et al., 2010) to age, sex, ethnicity, culture, socioeconomic status, and other socio-demographic factors (Anders et al., 2014; Knutson, 2013) as well as attitudes. Attitude is a psychological tendency that reflects the evaluation of something (i.e., a person, object, event, idea) with a certain degree of favor or disfavor (Eagly & Chaiken, 1993, as cited in Eagly & Chaiken, 2007). Attitudes are posited to derive from past experiences, emotions, and behaviors, in order to provide a quick assessment of the target object. Attitudes serve as guides and influencers of behavior and, conversely, they can be shaped by events and behaviors, maintaining a tendency to remain consistent and stable over time (*APA Dictionary of Psychology*, n.d.). Typically, attitudes encompass three components: cognitive, affective, and behavioral (Eagly & Chaiken, 2007). They have been demonstrated to impact sleep behaviors, either promoting or hindering sleep quality and hygiene across different populations, sexes, and age groups (Hamilton et al., 2021; Hertenstein et al., 2015; Morin et al., 2002; Zendels et al., 2021; Zhang et al., 2020). Attitudes can be assessed using self-report instruments, such as scales, while sleep can be assessed not only through self-report instruments but also through objective measures, such as actigraphy records and polysomnography (the gold standard sleep measure; Vlahoyiannis et al., 2020). Self-report instruments provide a cost-effective and fast way to collect sleep data, but they may be influenced by subjective bias. Polysomnography and other objective measures provide more robust data but are expensive and more cumbersome to use in data collection, namely for large epidemiological studies.

There are over 100 self-report instruments assessing sleep and sleep problems (Shahid et al., 2012). Some instruments, such as the Epworth Sleepiness Scale (Johns, 1991), only ask about sleep propensity across the day (e.g., *how likely are you to fall asleep while watching TV*) while others, like the Karolinska Sleep Questionnaire (KSQ; Nordin et al., 2013), combine sleep behaviors (e.g., *repeated awakenings*) with attitudinal items (e.g., *to what extent is sleep a problem*). The Dysfunctional Beliefs and Attitudes about Sleep scale (DBAS; Morin, 1993, 1994 as cited in Morin et al., 2002) is a self-report instrument of 30 items, expressing different insomnia-related dysfunctional beliefs and attitudes, that uses a 100-mm visual analog scale, to evaluate the level of agreement. This scale is widely used in insomnia research and has been translated and validated in several languages and cultures, including Portuguese (Clemente et al., 2023). The DBAS assesses faulty beliefs and attitudes, rather than evaluating sleep itself in terms of positivity or negativity. The Charlotte Attitude Toward Sleep scale (CATS; Peach & Gaultney, 2016) was developed to fill this need. This scale was designed to assess how sleep is perceived positively or negatively in daily life through 10 items using a 7-point Likert scale. The items are equally distributed in two subscales. The Time Commitments subscale measures sleep attitudes related to the time investment required by sleep and that may determine the overall favorable or unfavorable evaluation of time required to sleep (e.g., *I often pick other activities over going to bed early*). The Benefits subscale measures attitudes related to the pleasurable and restful benefits of sleep. (e.g., *I enjoy a good night’s sleep*).

CATS was initially developed to assess sleep attitudes in college students (Peach & Gaultney, 2016) but it has also been adapted to the general population (Ruggiero et al., 2019). CATS has been used to investigate demographic variations in sleep attitudes and their impact on sleep hygiene and outcomes (Ruggiero et al., 2019); explore the effects of sex differences on sleep attitudes, behaviors, and outcomes (Zendels et al., 2021); and examine the relationship between sleep irregularity and sleep attitudes in undergraduate students (Windred et al., 2021).

The main objective of this study was to translate the CATS into European Portuguese and gather evidence of its validity in a Portuguese sample. We hypothesized that the Portuguese version of CATS would show acceptable fit and reliability in the model sample and achieve at least configural invariance with the validation sample, with good average variance extracted (AVE) and discriminant validity between factors. CATS is a recent scale, on which very little research has been conducted, so far only the original study and Ruggiero et al. (2019) are published as far as we know. Thus, gathering further evidences of validity related to the internal structure and relations with other variables is an important topic for sleep research. To that end, we explore CATS relationships with other variables, under the general hypothesis that more positive attitudes toward sleep should predict sleep behaviors associated with better sleep hygiene. Thus, we hypothesize that the CATS factors to be positive predictors of the average weekly sleep duration (Sleep Dur_week_; calculated on the basis of reported sleep onset and wake time for working and non-working days, assuming a standard week with five working days and two non-working days), with subjective sleep quality, and with the number of hours of sleep people report they need, all variables taken from the KSQ. Finally, we tested the divergent validity between the CATS and KSQ factors. KSQ measures potentially problematic sleep behaviors and symptoms, while the CATS is a non-dysfunctional sleep attitudes. Therefore, we hypothesize that their correlation would be low to nonexistent.

## Methods

### Participants and procedures

We collected two subsamples, recruited by snowball non-probabilistic methods through social media channels, that were combined into a global sample. The protocols for the research questionnaires were presented online on the Qualtrics platform, made available via web link, and the order of the scales was randomized; all participants gave their informed consent before starting the questionnaires, and no form of incentive was offered. Subsample 1 was collected from the Portuguese general population; the 733 participants were aged 18 to 85 years of age (M = 40.34, SD = 14.1), mostly identifying as female (69.5%; 29.9% males; one participant identified with none of the sexes and was treated as a missing value for this variable). Exclusion criteria were being a minor (i.e., being less than 18 years old) and not having enough understanding of the Portuguese language to be able to answer the questionnaire. Subsample 2 was composed of 1125 higher education students aged 18 to 66 years old (M = 22.24; SD = 5.98); 63.3% participants identified as females, 9.8% as males, and 26.6% did not answer (4 identified as others and were treated as missing values). Inclusion criterion was to be enrolled in any year of a course leading to a university degree in Portugal; exclusion criteria were being under 18 years of age and having a proficiency in Portuguese that would prevent understanding the research protocol.

The two subsamples were combined for a total of 1858 participants, aged 18 to 85 years (M = 30.72, SD = 13.9), 69.5% of which were females, 14.2% were males with 16.4% missing responses. The file was randomly divided into two parts: one was labeled the test sample and was used to develop the CATS model; the other was designated as the validation sample and was used to test the model for measurement invariance. The model sample comprised 928 participants (68.9% female; average age = 30.59, SD = 13.77); the validation sample had 930 participants (70% female; average age = 30.85, SD = 14.12). For further details in sample description see supplementary tables in the annex.

This project is part of a larger study focused on sleep habits and burnout in higher education students and was approved by the Comissão de Ética do Instituto Universitário de Ciências Psicológicas, Sociais e da Vida (ISPA’s ethics committee) with the number D-038–06-2021.

### Instruments

The Charlotte Attitudes Toward Sleep—CATS (Peach & Gaultney, 2016) is a bi-dimensional scale measuring normal, non-dysfunctional, attitudes toward sleep. The Benefits subscale has five items and measures attitudes related to the enjoyment and restful benefits of sleep (e.g., *getting a good night’s sleep makes me happy*). The Time Commitments subscale evaluates attitudes about the positive or negative impact of the time that sleep requires (e.g., *I am inclined to skip sleep in order to socialize longer*) and has also five items, which should be reverse coded so that a higher score represents a more favorable attitude toward sleep. All items use a 7-point Likert-type measure. The final confirmatory factorial analysis in the model development (Peach & Gaultney, 2016, p. 27) showed the two-factor model to have a good fit [CFI = 0.94; TLI = 0.92; RMSEA = 0.07]; Benefits had a Cronbach *α* of 0.80 and Time Commitments an *α* of 0.77. The score of each subscale is calculated by averaging the five items within each subscale, and total score for the scale can be computed by averaging the ten items; in both cases a higher score expresses more positive attitudes toward sleep. Initially developed to be used with higher education students, displaying adequate sources of validity and reliability (Peach & Gaultney, 2016), the scale was adapted to adult working populations (Ruggiero et al., 2019). CATS contributed to illustrating the interaction between sleep attitudes and sex in predicting sleep hygiene and sleep quality (Zendels et al., 2021). Additionally, it revealed a positive association between Time Commitments and sleep regularity in higher education students (Windred et al., 2021).

The Karolinska Sleep Questionnaire (KSQ; Nordin et al., 2013) is a widely used questionnaire for the diagnose and research of sleep disturbances and problems mainly related to symptoms of insomnia, sleep apnea, and excessive sleepiness. It is composed by 18 items using a 6-point Likert scale and assessing frequency of sleep events (e.g., *difficulty falling asleep, gasping for air during sleep*), followed by several questions exploring sleep habits (e.g., sleep onset and wake up time in workdays and free days), nap habits, chronotype preference and perceptions of sleep quality and of sleep as a problem. Despite its popularity, KSQ psychometric properties are rarely assessed or reported. Nordin et al. (2013) is, to our best knowledge, the only peer-reviewed paper exclusively dedicated to evaluating different sources of validity evidence and reliability indicators of the KSQ. They settled in a 3-factor model (sleep quality, non-restorative sleep, sleep apnea) that uses 10 out of the 18 items of the scale part of the KSQ. This model had a good fit [RMSEA = 0.08; CFI = 0.94; GFI = 0.94], with Cronbach’s *α* ranging from 0.73 to 0.87 for the different factors across different sample groups. There are no (published) psychometric studies of the KSQ with Portuguese samples. We are currently conducting those studies based on the samples used in this paper and following a similar methodology. Our results show a good fit of the three-factor model, with the loss of item 4 in the sleep apnea factor (Tecedeiro et al., 2023).

### Scale translation and adaptation

Authorizations to use CATS and KSQ were obtained from their respective authors. CATS was translated into Portuguese by three independent translators with high fluency in English, and the translations were compared and merged by the authors of the article to create a consensual version. This version was back translated into English by a person who is a native speaker in both languages. No relevant differences appeared, and this version was used to explore validity based on response processes (American Educational Research Association et al., 1999, p. 12). Twenty-five individuals were asked to read the items aloud, explain what they thought was being queried, and verbalize their thought process while answering each item. No item displayed even minor comprehension issues, and it was consistently evident that participants were drawing upon their sleep attitudes and experiences to answer them.

### Data analysis

Statistical analysis was conducted using SPSS 29.0.0.0, Jamovi 2.3.21 and RStudio 2023.06.0Build421 software with R 4.3.1. Missing values were not replaced since they could not be assumed to be missing at random and were removed listwise (Peng et al., 2023). The criteria for items non-severe violation to a normal distribution that would recommend against confirmatory factor analysis (CFA) methods were absolute values of skewness (Sk) smaller or equal to 3 and kurtosis (Ku) smaller or equal to 7 (Finney & DiStefano, 2013; Marôco, 2021). To evaluate sources of evidence based on internal structure (American Educational Research Association et al., 2014), we conducted structural equation models (SEM) of CFA of the original scale factor structures using a robust maximum likelihood method (MLR) with Satorra-Bentler correction using the SEMLj module (Gallucci & Jentschke, n.d.), a jamovi interface to lavaan R package (Rosseel 2012), since items are ordinal with violations of normality assumptions. Goodness of fit criteria indicative of good fit of the model to the data were a robust root mean square of approximation (RMSEA) lower or equal to 0.08, or a robust standardized root mean square residual (SRMS) lower or equal to 0.08, a comparative fit index (CFI) equal or higher than 0.9, and a Tucker Lewis Index (TLI) equal or higher than 0.9 (Hu & Bentler, 1999; Marôco, 2021). Criteria for adequate item loading was a standardized factor loading (*β*) equal to or higher than 0.5. Reliability, based on internal consistence assessment, was estimated through Cronbach’s *α* and McDonalds *ώ* (Hayes & Coutts, 2020) with 0.7 or higher as criterion for acceptable reliability. As a criterion for convergence validity, the minimal average variance extracted (AVE) was set at 0.5 (Henseler, 2017; Marôco, 2021). Discriminant evidence of a factor was established by an AVE higher than the squares of its correlations with other factors (Marôco, 2021), or by a heterotrait/monotrait ratio of correlations (HTMT) higher that 0.85 (Henseler, 2017). Measurement invariance between samples (test vs validation; males vs females) was tested by imposing further constraints on the free (configural) model: factor loadings (metric invariance), factor loadings plus intercepts (scalar invariance), and factor loadings plus intercepts plus residues (strict invariance; Marôco, 2021; Putnick & Bornstein, 2016); invariance criteria was a non-significant difference between the χ^2^ of two consecutive nested models, or a decrease in CFI smaller than 0.01 or an increase in RMSEA larger than 0.02. Evidence based on relations to other variables and test-criterion evidence (American Educational Research Association et al., 2014) were estimated through SEM causal models using the MLR method. Statistical significance was considered to be *p* < 0.05 for all analysis.

## Results

### Item sensitivity

Descriptive statistics for the CATS items in the global sample are given in Table 1. All scale values were observed in all items, with medians varying between 2 and 7. Based on the Sk and Ku values, items 1, 3, 8, and 9, all belonging to the Benefits factor, exhibit significant deviations from normal distributions.

**Table 1:** Descriptive statistics for the CATS items in the global sample

Items	*N*		Median	Mode	Skewness	Skewness std. error	Kurtosis	Kurtosis std. error	Minimum	Maximum
	Valid	Missing								
CATS_1	1727	131	7.00	7.00	− 5.17	0.06	36.40	0.12	1.00	7.00
CATS_2*	1727	131	3.00	3.00	0.21	0.06	− 1.24	0.12	1.00	7.00
CATS_3	1727	131	7.00	7.00	− 2.17	0.06	6.27	0.12	1.00	7.00
CATS_4*	1727	131	3.00	2.00	0.42	0.06	− 1.08	0.12	1.00	7.00
CATS_5	1727	131	6.00	7.00	− 1.34	0.06	1.03	0.12	1.00	7.00
CATS_6*	1727	131	5.00	6.00	− 0.29	0.06	− 1.25	0.12	1.00	7.00
CATS_7*	1727	131	3.00	2.00	0.25	0.06	− 1.25	0.12	1.00	7.00
CATS_8	1727	131	7.00	7.00	− 2.63	0.06	9.14	0.12	1.00	7.00
CATS_9	1727	131	7.00	7.00	− 3.42	0.06	17.42	0.12	1.00	7.00
CATS_10*	1727	131	4.00	6.00	− 0.10	0.06	− 1.36	0.12	1.00	7.00

^*^Reverse coded item

The model sample encountered similar normality issues with items 1, 8, and 9, with item 9 having no responses in the “totally disagree” option. Likewise, the validation sample experienced identical normality issues with the same items, and item 1 had no responses in the “partially disagree” option. Medians for both samples varied between 2 and 7.

### Validity evidence based on internal structure

The initial CFA was conducted on the model sample, using the original factor structure of the scale (Peach & Gaultney, 2016). Items 2, 5, and 6 had factor loadings (*β*s) smaller than 0.5. They were excluded from the model and the CFA was conducted again. In this revised analysis, all items had *β*s higher than 0.5, and the model had an excellent fit [robust RMSEA = 0.043; robust SMRS = 0.026; robust CFI = 0.987; TLI = 0.979]. The two factors had good reliability [Benefits: *α* = 0.775 and *ώ*^1^ = 0.790; Time Commitments: *α* = 0.759 and *ώ*^1^ = 0.764]. AVE was good for Time Commitments [0.522] and acceptable for Benefits [0.497]. Correlation between the two factors was 0.0128; its square value (0.0196) was smaller than the factors’ AVEs and the HTMT ratio of correlation was 0.17. The existence of discriminant validity argues against a second order factor and the calculation of a global score, which we did not use in this study.

We tested this model for measurement invariance between the test and validation samples. The free (no constraints) model showed configural invariance with an excellent fit [robust RMSEA = 0.039; robust SRMR = 0.024; robust CFI = 0.990; robust TLI = 0.983]. We then imposed further constraints on the model, as described above. Table 2 summarizes the results, showing that this model achieves strict invariance using the ∆CFI and ∆RMSEA criteria.

**Table 2:** Satorra-Bentler χ^2^ differences between model and test sample invariance models

Invariance type	Constraints	SBχ^2^	∆ χ^2^	DF	∆ DF	*p*	Robust CFI	∆ Robust CFI	Robust RSMEA	∆ Robust RMSEA
Configural invariance	No constraints	68.53		26			0.988		0.039	
Metric invariance	Loadings	71.13	0.870	31	5	0.927	0.993	− 0.003	0.029	− 0.010
Scalar invariance	Previous + intercepts	86.42	15.410	36	5	0.009	0.990	0.003	0.033	0.004
Strict invariance	Previous + residuals	133.83	8.263	43	7	0.310	0.988	0.002	0.032	− 0.001

Table 3 shows the reliability and discriminant validity indicators of the two-factor CATS model on the test and validation samples.

**Table 3:** Reliability and AVE indicators of two-factor model across samples

Sample	Factor	*α*	*ω*_1_	AVE	Inter factor *r*/*r*^2^	HTMT
Test sample	Benefits	0.775	0.794	0.505	0.147/0.021	0.146
Test sample	Time Commitments	0.759	0.764	0.521		
Validation sample	Benefits	0.777	0.788	0.496	0.109/0.012	
Validation sample	Time Commitments	0.781	0.784	0.550		

We also tested for measurement invariance between sexes in the global sample. The free model had an excellent fit [robust RMSEA = 0.040; robust SRMR = 0.024; CFI = 0.987; TLI = 0.979]. The imposition of further constraints established that the model achieves strict invariance between sexes using the ∆CFI and ∆RMSEA criteria (Table 4).

**Table 4:** Satorra-Bentler χ^2^ differences between sexes invariance models

Invariance type	Constraints	SBχ^2^	∆ χ^2^	DF	∆ DF	*p*	Robust CFI	∆ Robust CFI	Robust RSMEA	∆ Robust RMSEA
Configural invariance	No constraints	65.07		26			0.988		0.042	
Metric invariance	Loadings	72.98	3.62	31	5	0.606	0.989	0.001	0.037	− 0.005
Scalar invariance	Previous + intercepts	96.49	23.39	36	5	< 0.001	0.982	− 0.007	0.043	0.006
Strict invariance	Previous + residuals	143.83	9.408	43	7	0.225	0.978	− 0.004	0.044	0.001

Table 5 presents the reliability and discriminant validity indicators in the strict invariance model between samples.

**Table 5:** Reliability and AVE indicators of the invariance model between sexes

Sample	Factor	*α*	*ω*_1_	AVE	Inter factor *r*/*r*^2^	HTMT
Model sample	Benefits	0.755	0.767	0.467	0.155/0.024	0.163
Model sample	Time Commitments	0.772	0.774	0.736		
Validation sample	Benefits	0.796	0.834	0.571	0.136/0.018	
Validation sample	Time Commitments	0.773	0.778	0.541		

**Fig. 1:**
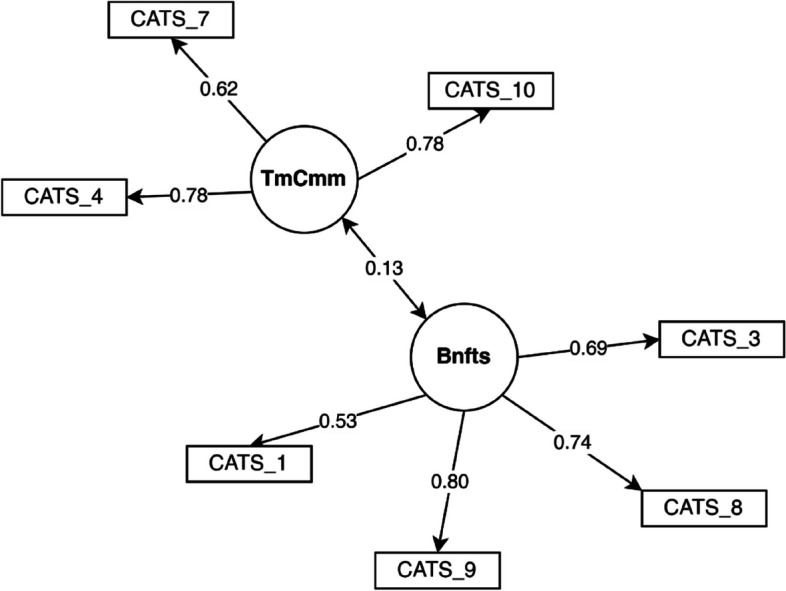
CATS model fitted in the global sample

Finally, we fitted the model to the global sample (Fig. 1), so that we could use CATS factors in causal models (see next section).

The model had an excellent fit [robust RMSEA = 0.043; robust SRMR = 0.021; robust CFI = 0.988; robust TLI = 0.980]. Table 6 shows the reliability indicators and AVE of each factor, with good reliability and acceptable convergent validity in each factor.

**Table 6:** Reliability indicators and AVEs of CATS factors in the global sample

Factors	*α*	*ω*_1_	*ω*_2_	*ω*_3_	AVE
Benefits	0.776	0.791	0.791	0.792	0.493
Time Commitments	0.770	0.774	0.774	0.775	0.536

Correlation between factors was 0.130 (*r*^2^ = 0.169) and the HTMT ratio was 0.146, both indicating discriminant validity between factors. Table 7 presents the descriptive statistics for the averaged raw scores of each factor.

**Table 7:** Descriptive statistics of CATS factor raw scores

Factors	*N*	Missing	Median	Mode	Minimum	Maximum	Skewness	Kurtosis	Percentiles		
									**25th**	**50th**	**75th**
Benefits	1727	131	6.75	7	1	7	− 2.808	13.557	6.25	6.75	7
Time Commitments	1727	131	3.67	2	1	7	0.214	− 0.915	2.33	3.67	5

### Validity evidence based on relationships with other variables

**Fig. 2:**
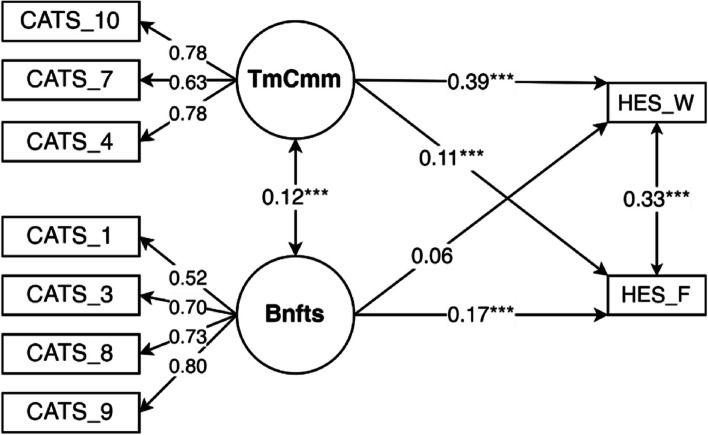
Causal model of the influence of CATS factor over hours of sleep on free days (HES_F) and workdays (HES_W) (***p < 0.001)

To assess concurrent criterion validity, we created a hypothetical causal model to test how well CATS factors can predict the Sleep Dur_week_. This model had an excellent fit [robust RMSEA = 0.047; robust SMRM = 0.025] and fit indexes [robust CFI = 0.981; robust TLI = 0.970]. Benefits *β* was 0.107 [*p* = 0.001] and Time Commitments *β* was 0.362 [*p* < 0.01]. Considering the low *β* of Benefits, we decided to further explore the predictive power of CATS over the sleep duration on workdays and free days, the two components of Sleep Dur_week_ (Fig. 2).

The model had a fit similar to the previous one [robust RMSEA = 0.046; robust SMRM = 0.027; robust CFI = 0.978; robust TLI = 0.965].

Another causal model was used to estimate the influence of CATS factors on how many hours of sleep people say they need. The model’s fit was excellent, with excellent indicators [robust RMSEA = 0.041; robust SMRM = 0.022; robust CFI = 0.985; robust TLI = 0.976]. Benefits’ *β* was 0.239 [*p* < 0.001] and Time Commitments had a *β* of 0.058 [*p* = 0.042].

A causal model was also used to test the influence of CATS factors on subjective sleep quality, reported on the KSQ (this question is coded so that a higher score expresses a perception of better sleep quality). Fit indicators were excellent [robust RMSEA = 0.042; robust SMRM = 0.022; robust CFI = 0.984; robust TLI = 0.975]. Benefits had a *β* of − 0.040 (*p* = 0.177) and Time Commitments’ *β* was − 0.298 (*p* < 0.001).

Finally, we evaluated the divergent validity between CATS and KSQ factors by creating an MRL measurement model with both scales. The model had an excellent fit [robust RMSEA = 0.056; robust SMRM = 0.036; robust CFI = 0.960; robust TLI = 0.949]. Table 8 presents the AVEs of the KSQ factors, HTMT ratios, and the correlations/squared correlations with the CATS factors, showing a good divergent validity between tests.

**Table 8:** KSQ AVEs, HTMT ratios, and correlations between KSQ and CATS factors

KSQ factors	AVE	Benefits		Time Commitments	
		**HTMT**	***r*/*r***^**2**^	**HTMT**	***r*/*r***^**2**^
Sleep quality	0.568	0.057	− 0.042/0.002	0.299	− 0.322/0.104
Non-restorative sleep	0.694	0.048	0.032/0.001	0.460	− 0.415/0.172
Sleep apnea symptoms	0.748	0.037	− 0.026/0.001	0.214	0.223/0.050

## Discussion

This study examines CATS’ psychometric properties in Portuguese samples. CATS’ two-factor structure had an excellent fit and had strict invariance of measure between model and validation samples as well as between sexes, but at the cost of three items: item 5 (*I look forward to a full night of sleep*) from the Benefits factor and items 2 (*I am inclined to skip sleep in order to socialize longer*) and 6 (*In the past, I haven’t made enough time for adequate sleep in my schedule*) from the Time Commitments factor, all due to low factor weights (*β*). Items 1 and 5 had already shown sensitivity problems with a strong deviation from normality, a problem shared by item 8 (*Getting a full night of sleep is satisfying to me*) and item 9 (*I enjoy a good night’s sleep*). In consequence, Benefits raw scores had a strong deviation from a normal distribution, a sign of poor sensitivity, clearly made visible by a score of 6.25 at the 25th percentile. This may explain why the AVEs of the factor was always very close to the convergent validity criterion (≥ 0.5) and why the factor was not a strong predictor of sleep behaviors. Nonetheless, the scale displayed strong factorial validity, good reliability, acceptable convergent validity of each factor, and good discriminant validity between factors. In sources of evidence relating to other variables, Benefits was a weak, but statistically significant, predictor of hours slept on free days but not on workdays, as well as a weak predictor of the number of hours people report they need; it was, however, a poor predictor of subjective sleep quality. On the other hand, Time Commitments was a moderate predictor of the average weekly sleep duration, particularly on work days, i.e., higher scores of Time Commitments lead to longer sleep duration especially on workdays; this relationship requires further study and exploration, including with clinical samples; the factor was not a good predictor of the reported sleep needed, suggesting an independence of the two constructs, i.e., the fact that one prioritizes sleep does not imply the estimation that more sleep hours are needed; the factor was also a moderate but negative predictor of subjective sleep quality, indicating that a more positive view of the time invested in sleep is associated with lower subjective sleep quality. These results are contrary to the ones published by Wildred et al. (2021) who found that CATS Benefits were positively associated with sleep quality suggesting that people that report sleep as an enjoyable or beneficial activity tend to have a better sleep quality. On the other hand, sleep regularity was associated with CATS Time Commitments but not with CATS Benefits. The fact that we had a negative association between Time Commitments and sleep quality might suggest that the individuals reporting a lower sleep quality might value the time dedicated for sleep even more than the good sleepers. Further studies assessing other metrics associated with sleep quality like sleep regularity or fragmentation should be studied in future studies. The current results align with other research findings where social timing and shorter sleep duration was associated with a poorer sleep quality in individuals of the general population and patients with sleep disorders (Reis et al., 2020), for these subjects not being able to sleep enough on workdays due to social constraints make them reporting poor sleep. These results also call for further study, both in general population and in clinical samples, particularly with insomnia and delayed sleep–wake phase disorder patients assessing CATS. Finally, CATS showed good divergent validity with the KSQ, as predicted, showing that scale results are not contaminated by potential sleep problems, but again it will be important to test this relationship in clinical samples.

The internal consistency of the current study is similar to the one published for the total scale using a sample of adults from the general population (*α* = 0.79) (Ruggiero et al., 2019) as well as the one published for the original validation study with college students, which like our study, validated the two-factor structure (*α* total = 0.75, *α* Benefits = 0.80, *α* Time Commitments = 0.77) (Peach & Gaultney, 2016), but lower than other study with college students (*α* = 0.86) (Peach et al., 2018). To the best of our knowledge, these are the only studies published on the psychometric properties of data gathered with CATS. Authors have suggested a single factor and/or a two-factor structure for CATS. Our analysis on the discriminant validity of the Benefits and Time Commitments found no empirical support for the single factor structure. Since Benefits and Time Commitments are measuring different things, a CATS global score is not defensible. Further studies should be conducted to evaluate evidences of validity related to the internal structure, including not only internal consistency, but also other metrics, e.g., relationships with other variables, response processes, etc., to assess CATS psychometric validity and reliability.

This study has some limitations. We used convenience samples collected over social media, with a disproportionally large number of women, and a relevant number of missing values. The option to remove missing values list-wise meant that statistical power of the analysis was lower than possible, but not low enough to weaken the results. Despite the sex gap, we were able to show that the scale was invariant between sexes, but we opted not to report sex raw scores of CATS factors due to the imbalance. We did not probe for temporal stability of the factors, a relevant analysis, since attitudes should be, by definition, stable constructs over time. We also did not have the opportunity to use the scale in clinical samples, namely in patients with insomnia, an important aspect in a non-dysfunctional attitudes construct. Despite the limitations previously mentioned, we believe the CATS is a valuable instrument. It enables the assessment of an important and often overlooked dimension of sleep—attitudes—which has garnered significant interest in the sleep field due to its relevance for sleep health.

## Conclusions

The CATS scale assesses non-dysfunctional attitudes toward sleep, with excellent sources of evidence related to response processes and internal structured, and somewhat mixed results on sources of evidence related to relationships with other variables. We found CATS to be a relevant and promising scale for sleep research studies and interventions; it requires, however, further work at item level. Further research should focus on revising/rewording items to increase sensitivity and predictive value, followed by the reassessment of its psychometric qualities in large, sex balanced, samples. Once the sensitivity issue is solved, it would be important to test invariance with other Portuguese-speaking countries (e.g., Brazil, Angola, Mozambique). Moreover, it would also be important to explore construct validity of this instrument in samples of sleep disorder patients, namely patients with insomnia or delayed sleep–wake phase disorder.

## Supplementary Information

Supplementary Material 1.

## Data Availability

The datasets generated and/or analyzed during the current study are not publicly available due to academic considerations but are available from the corresponding author on reasonable request.

## Abbreviations

AVE: Average variance extracted
CATS: Charlotte Attitudes Toward Sleep scale
CFA: Confirmatory factor analysis
CFI: Comparative fit index
DBAS: Dysfunctional Beliefs and Attitudes about Sleep scale
HTMT: Heterotrait/monotrait ratio of correlations
KSQ: Karolinska Sleep Questionnaire
MLR: Robust maximum likelihood method
RMSEA: Root mean square of approximation
SEM: Structural equation model
Sleep Dur_week_: Average weekly sleep duration
SRMR: Standardized root mean square residual
TLI: Tucker Lewis Index
