# Supplementary material for: Assessing non-dysfunctional attitudes toward sleep: psychometric properties of the Charlotte Attitudes Toward Sleep scale in Portuguese samples
Source: Psicol Reflex Crit. 2024 Sep 14;37:36. doi: 10.1186/s41155-024-00320-3 (PMC11401811; doi:10.1186/s41155-024-00320-3)
Supplement: Supplementary file 1 — Supplementary Material 1. [file 41155_2024_320_MOESM1_ESM.pdf]

Frequencies

Statistics

	N		Mean	Median	Mode	Std. Deviation
	Valid	Missing				
Genero	1554	304	,17	,00	0	,375
Idade	1548	310	30,72	23,00	18	13,943
KSQhorasdormir_WD_numerico	1769	89	11,6227	3,5000	23,00	11,06095
KSQhorasdormir_FD_numerico	1764	94	7,2147	1,8750	,00	9,74962
KSQhorasacordar_WD_decimal	1770	88	7,5098	7,5000	7,00	1,17396
KSQhorasacordar_FD_Decimal	1764	94	9,5865	9,5000	9,00	1,52486
KSQlatenciasono_WD_Decimal	1781	77	,5132	,3333	,50	,47127
KSQlatenciasono_FD_Decimal	1775	83	,5583	,3333	,50	,57721
HorasEfectivasSono_WD_decimal	1759	99	7,3532	7,5000	8,00	1,37064
HorasEfectivasSono_FD_decimal	1750	108	8,6498	8,6667	8,00	1,45909
KSQ_WeekAverageSleepDuration	1750	108	7,7057	7,8024	8,29	1,23095
KSQqualidadesono	1791	67	2,69	3,00	2	1,003

Statistics

	Skewness	Std. Error of Skewness	Kurtosis	Std. Error of Kurtosis	Minimum
Genero	1,766	,062	1,120	,124	0
Idade	1,050	,062	,074	,124	18
KSQhorasdormir_WD_numerico	,026	,058	-1,982	,116	,00
KSQhorasdormir_FD_numerico	,963	,058	-1,023	,116	,00
KSQhorasacordar_WD_decimal	1,166	,058	4,138	,116	3,50
KSQhorasacordar_FD_Decimal	,250	,058	,067	,116	4,50
KSQlatenciasono_WD_Decimal	2,116	,058	5,606	,116	,00
KSQlatenciasono_FD_Decimal	2,304	,058	6,399	,116	,00
HorasEfectivasSono_WD_decimal	-,424	,058	1,431	,117	2,00
HorasEfectivasSono_FD_decimal	-,093	,059	1,129	,117	2,50
KSQ_WeekAverageSleepDuration	-,695	,059	3,384	,117	,00
KSQqualidadesono	,348	,058	-,537	,116	1

Statistics

	Maximum
Genero	1
Idade	85
KSQhorasdormir_WD_numerico	23,98
KSQhorasdormir_FD_numerico	23,98
KSQhorasacordar_WD_decimal	16,00
KSQhorasacordar_FD_Decimal	15,50
KSQlatenciasono_WD_Decimal	3,50
KSQlatenciasono_FD_Decimal	4,00
HorasEfectivasSono_WD_decimal	14,00
HorasEfectivasSono_FD_decimal	14,50
KSQ_WeekAverageSleepDuration	13,14
KSQqualidadesono	5

Frequency Table

Genero

		Frequency	Percent	Valid Percent	Cumulative Percent
Valid	Feminino	1291	69,5	83,1	83,1
	Masculino	263	14,2	16,9	100,0
	Total	1554	83,6	100,0	
Missing	System	304	16,4		
Total		1858	100,0		

Idade

		Frequency	Percent	Valid Percent	Cumulative Percent
Valid	18	170	9,1	11,0	11,0
	19	164	8,8	10,6	21,6
	20	156	8,4	10,1	31,7
	21	142	7,6	9,2	40,8
	22	98	5,3	6,3	47,2
	23	71	3,8	4,6	51,7
	24	44	2,4	2,8	54,6
	25	27	1,5	1,7	56,3
	26	28	1,5	1,8	58,1
	27	16	,9	1,0	59,2
	28	11	,6	,7	59,9
	29	25	1,3	1,6	61,5
	30	15	,8	1,0	62,5
	31	10	,5	,6	63,1
	32	13	,7	,8	64,0
	33	13	,7	,8	64,8
	34	17	,9	1,1	65,9
	35	15	,8	1,0	66,9
	36	18	1,0	1,2	68,0
	37	9	,5	,6	68,6
	38	29	1,6	1,9	70,5
	39	25	1,3	1,6	72,1
	40	26	1,4	1,7	73,8
	41	20	1,1	1,3	75,1
	42	29	1,6	1,9	76,9
	43	23	1,2	1,5	78,4
	44	18	1,0	1,2	79,6
	45	37	2,0	2,4	82,0
	46	31	1,7	2,0	84,0
	47	19	1,0	1,2	85,2
	48	16	,9	1,0	86,2

Idade

		Frequency	Percent	Valid Percent	Cumulative Percent
	49	12	,6	,8	87,0
	50	23	1,2	1,5	88,5
	51	18	1,0	1,2	89,7
	52	19	1,0	1,2	90,9
	53	16	,9	1,0	91,9
	54	10	,5	,6	92,6
	55	7	,4	,5	93,0
	56	14	,8	,9	93,9
	57	9	,5	,6	94,5
	58	7	,4	,5	95,0
	59	9	,5	,6	95,5
	60	6	,3	,4	95,9
	61	8	,4	,5	96,4
	62	4	,2	,3	96,7
	63	11	,6	,7	97,4
	64	4	,2	,3	97,7
	65	6	,3	,4	98,1
	66	5	,3	,3	98,4
	67	9	,5	,6	99,0
	68	4	,2	,3	99,2
	69	2	,1	,1	99,4
	71	3	,2	,2	99,5
	72	1	,1	,1	99,6
	74	1	,1	,1	99,7
	75	1	,1	,1	99,7
	76	1	,1	,1	99,8
	78	1	,1	,1	99,9
	83	1	,1	,1	99,9
	85	1	,1	,1	100,0
	Total	1548	83,3	100,0	
Missing	System	310	16,7		
Total		1858	100,0		

KSQhorasdormir_WD_numerico

		Frequency	Percent	Valid Percent	Cumulative Percent
Valid	,00	374	20,1	21,1	21,1
	,17	3	,2	,2	21,3
	,25	9	,5	,5	21,8
	,50	124	6,7	7,0	28,8
	,67	2	,1	,1	28,9
	,75	5	,3	,3	29,2
	,83	1	,1	,1	29,3
	1,00	189	10,2	10,7	40,0
	1,50	48	2,6	2,7	42,7
	1,73	1	,1	,1	42,7
	2,00	88	4,7	5,0	47,7
	2,25	1	,1	,1	47,8
	2,50	12	,6	,7	48,4
	3,00	25	1,3	1,4	49,9
	3,50	5	,3	,3	50,1
	3,75	1	,1	,1	50,2
	4,00	5	,3	,3	50,5
	4,50	2	,1	,1	50,6
	6,00	2	,1	,1	50,7
	7,50	1	,1	,1	50,8
	17,00	1	,1	,1	50,8
	18,00	1	,1	,1	50,9
	19,00	4	,2	,2	51,1
	19,50	2	,1	,1	51,2
	20,00	6	,3	,3	51,6
	20,50	1	,1	,1	51,6
	20,75	1	,1	,1	51,7
	21,00	16	,9	,9	52,6
	21,33	1	,1	,1	52,6
	21,50	15	,8	,8	53,5
	21,67	1	,1	,1	53,5
	21,83	1	,1	,1	53,6
	22,00	94	5,1	5,3	58,9
	22,25	2	,1	,1	59,0
	22,50	115	6,2	6,5	65,5
	22,62	1	,1	,1	65,6
	22,67	1	,1	,1	65,6
	22,75	8	,4	,5	66,1
	23,00	384	20,7	21,7	87,8
	23,05	2	,1	,1	87,9
	23,25	2	,1	,1	88,0
	23,33	1	,1	,1	88,1

KSQhorasdormir_WD_numerico

		Frequency	Percent	Valid Percent	Cumulative Percent
	23,45	1	,1	,1	88,1
	23,50	186	10,0	10,5	98,6
	23,65	1	,1	,1	98,7
	23,67	4	,2	,2	98,9
	23,75	16	,9	,9	99,8
	23,83	2	,1	,1	99,9
	23,98	1	,1	,1	100,0
	Total	1769	95,2	100,0	
Missing	System	89	4,8		
Total		1858	100,0		

KSQhorasdormir_FD_numerico

		Frequency	Percent	Valid Percent	Cumulative Percent
Valid	,00	371	20,0	21,0	21,0
	,02	2	,1	,1	21,1
	,17	1	,1	,1	21,2
	,25	3	,2	,2	21,4
	,50	100	5,4	5,7	27,0
	,62	1	,1	,1	27,1
	,67	2	,1	,1	27,2
	,75	2	,1	,1	27,3
	,77	1	,1	,1	27,4
	,83	1	,1	,1	27,4
	1,00	318	17,1	18,0	45,5
	1,25	2	,1	,1	45,6
	1,33	2	,1	,1	45,7
	1,50	73	3,9	4,1	49,8
	1,67	2	,1	,1	49,9
	1,75	1	,1	,1	50,0
	2,00	209	11,2	11,8	61,8
	2,50	27	1,5	1,5	63,4
	3,00	94	5,1	5,3	68,7
	3,50	6	,3	,3	69,0
	3,67	1	,1	,1	69,1
	3,75	1	,1	,1	69,2
	4,00	34	1,8	1,9	71,1
	4,25	1	,1	,1	71,1
	4,50	2	,1	,1	71,3
	5,00	9	,5	,5	71,8
	5,50	1	,1	,1	71,8
	6,00	5	,3	,3	72,1

KSQhorasdormir_FD_numerico

		Frequency	Percent	Valid Percent	Cumulative Percent
	7,00	1	,1	,1	72,2
	12,00	1	,1	,1	72,2
	19,00	1	,1	,1	72,3
	20,00	4	,2	,2	72,5
	20,75	1	,1	,1	72,6
	21,00	15	,8	,9	73,4
	21,50	4	,2	,2	73,6
	22,00	67	3,6	3,8	77,4
	22,50	44	2,4	2,5	79,9
	22,67	1	,1	,1	80,0
	22,75	1	,1	,1	80,0
	23,00	232	12,5	13,2	93,2
	23,15	1	,1	,1	93,3
	23,25	3	,2	,2	93,4
	23,33	2	,1	,1	93,5
	23,50	101	5,4	5,7	99,3
	23,75	10	,5	,6	99,8
	23,83	2	,1	,1	99,9
	23,98	1	,1	,1	100,0
	Total	1764	94,9	100,0	
Missing	System	94	5,1		
Total		1858	100,0		

KSQhorasacordar_WD_decimal

		Frequency	Percent	Valid Percent	Cumulative Percent
Valid	3,50	1	,1	,1	,1
	4,00	2	,1	,1	,2
	4,50	2	,1	,1	,3
	4,75	1	,1	,1	,3
	5,00	14	,8	,8	1,1
	5,33	2	,1	,1	1,2
	5,50	11	,6	,6	1,9
	5,67	4	,2	,2	2,1
	5,75	7	,4	,4	2,5
	5,83	4	,2	,2	2,7
	5,92	2	,1	,1	2,8
	6,00	125	6,7	7,1	9,9
	6,12	1	,1	,1	9,9
	6,15	1	,1	,1	10,0
	6,17	7	,4	,4	10,4
	6,20	1	,1	,1	10,5
	6,25	22	1,2	1,2	11,7
	6,33	21	1,1	1,2	12,9
	6,42	1	,1	,1	12,9
	6,50	139	7,5	7,9	20,8
	6,58	1	,1	,1	20,8
	6,67	17	,9	1,0	21,8
	6,75	46	2,5	2,6	24,4
	6,83	26	1,4	1,5	25,9
	7,00	336	18,1	19,0	44,9
	7,08	2	,1	,1	45,0
	7,17	12	,6	,7	45,6
	7,25	36	1,9	2,0	47,7
	7,33	24	1,3	1,4	49,0
	7,42	4	,2	,2	49,3
	7,50	220	11,8	12,4	61,7
	7,58	1	,1	,1	61,8
	7,67	14	,8	,8	62,5
	7,75	23	1,2	1,3	63,8
	7,83	12	,6	,7	64,5
	7,92	4	,2	,2	64,7
	8,00	272	14,6	15,4	80,1
	8,08	2	,1	,1	80,2
	8,17	4	,2	,2	80,5
	8,22	1	,1	,1	80,5
	8,25	14	,8	,8	81,3
	8,28	1	,1	,1	81,4

KSQhorasacordar_WD_decimal

		Frequency	Percent	Valid Percent	Cumulative Percent
	8,33	5	,3	,3	81,6
	8,50	91	4,9	5,1	86,8
	8,67	2	,1	,1	86,9
	8,75	7	,4	,4	87,3
	8,83	2	,1	,1	87,4
	9,00	98	5,3	5,5	92,9
	9,17	2	,1	,1	93,1
	9,25	1	,1	,1	93,1
	9,50	30	1,6	1,7	94,8
	9,75	3	,2	,2	95,0
	10,00	44	2,4	2,5	97,5
	10,25	1	,1	,1	97,5
	10,33	1	,1	,1	97,6
	10,50	10	,5	,6	98,1
	10,67	1	,1	,1	98,2
	11,00	17	,9	1,0	99,2
	11,50	8	,4	,5	99,6
	11,75	1	,1	,1	99,7
	12,00	2	,1	,1	99,8
	12,50	2	,1	,1	99,9
	16,00	2	,1	,1	100,0
	Total	1770	95,3	100,0	
Missing	System	88	4,7		
Total		1858	100,0		

KSQhorasacordar_FD_Decimal

		Frequency	Percent	Valid Percent	Cumulative Percent
Valid	4,50	1	,1	,1	,1
	5,00	3	,2	,2	,2
	5,83	1	,1	,1	,3
	6,00	9	,5	,5	,8
	6,25	1	,1	,1	,9
	6,33	1	,1	,1	,9
	6,50	12	,6	,7	1,6
	6,75	2	,1	,1	1,7
	7,00	68	3,7	3,9	5,6
	7,33	5	,3	,3	5,8
	7,50	61	3,3	3,5	9,3
	7,67	1	,1	,1	9,4
	7,75	6	,3	,3	9,7
	7,83	3	,2	,2	9,9
	8,00	198	10,7	11,2	21,1
	8,08	1	,1	,1	21,1
	8,17	1	,1	,1	21,2
	8,22	1	,1	,1	21,3
	8,25	4	,2	,2	21,5
	8,33	1	,1	,1	21,5
	8,50	104	5,6	5,9	27,4
	8,67	3	,2	,2	27,6
	8,75	5	,3	,3	27,9
	9,00	324	17,4	18,4	46,3
	9,25	1	,1	,1	46,3
	9,33	1	,1	,1	46,4
	9,50	113	6,1	6,4	52,8
	9,65	1	,1	,1	52,8
	9,67	1	,1	,1	52,9
	9,75	6	,3	,3	53,2
	9,83	1	,1	,1	53,3
	10,00	312	16,8	17,7	71,0
	10,25	4	,2	,2	71,2
	10,50	89	4,8	5,0	76,2
	10,75	2	,1	,1	76,4
	10,83	1	,1	,1	76,4
	11,00	194	10,4	11,0	87,4
	11,25	1	,1	,1	87,5
	11,50	32	1,7	1,8	89,3
	11,75	2	,1	,1	89,4
	11,83	1	,1	,1	89,5
	12,00	122	6,6	6,9	96,4

KSQhorasacordar_FD_Decimal

		Frequency	Percent	Valid Percent	Cumulative Percent
	12,17	1	,1	,1	96,4
	12,25	2	,1	,1	96,5
	12,50	15	,8	,9	97,4
	13,00	31	1,7	1,8	99,1
	13,50	2	,1	,1	99,3
	14,00	10	,5	,6	99,8
	15,00	2	,1	,1	99,9
	15,50	1	,1	,1	100,0
	Total	1764	94,9	100,0	
Missing	System	94	5,1		
Total		1858	100,0		

KSQlatenciasono_WD_Decimal

		Frequency	Percent	Valid Percent	Cumulative Percent
Valid	,00	15	,8	,8	,8
	,00	1	,1	,1	,9
	,02	4	,2	,2	1,1
	,03	7	,4	,4	1,5
	,05	3	,2	,2	1,7
	,08	139	7,5	7,8	9,5
	,12	5	,3	,3	9,8
	,13	3	,2	,2	9,9
	,15	1	,1	,1	10,0
	,17	281	15,1	15,8	25,8
	,20	2	,1	,1	25,9
	,23	2	,1	,1	26,0
	,25	289	15,6	16,2	42,2
	,27	5	,3	,3	42,5
	,28	3	,2	,2	42,7
	,30	1	,1	,1	42,7
	,32	1	,1	,1	42,8
	,33	165	8,9	9,3	52,0
	,37	10	,5	,6	52,6
	,38	1	,1	,1	52,7
	,40	1	,1	,1	52,7
	,42	18	1,0	1,0	53,7
	,50	5	,3	,3	54,0
	,50	371	20,0	20,8	74,8
	,58	2	,1	,1	75,0
	,67	33	1,8	1,9	76,8
	,70	2	,1	,1	76,9

KSQlatenciasono_WD_Decimal

		Frequency	Percent	Valid Percent	Cumulative Percent
	,75	61	3,3	3,4	80,3
	,83	1	,1	,1	80,4
	,87	2	,1	,1	80,5
	1,00	230	12,4	12,9	93,4
	1,08	1	,1	,1	93,5
	1,12	1	,1	,1	93,5
	1,17	1	,1	,1	93,6
	1,25	6	,3	,3	93,9
	1,33	1	,1	,1	94,0
	1,50	32	1,7	1,8	95,8
	2,00	63	3,4	3,5	99,3
	2,50	5	,3	,3	99,6
	3,00	6	,3	,3	99,9
	3,50	1	,1	,1	100,0
	Total	1781	95,9	100,0	
Missing	System	77	4,1		
Total		1858	100,0		

KSQlatenciasono_FD_Decimal

		Frequency	Percent	Valid Percent	Cumulative Percent
Valid	,00	30	1,6	1,7	1,7
	,02	3	,2	,2	1,9
	,03	8	,4	,5	2,3
	,05	4	,2	,2	2,5
	,08	151	8,1	8,5	11,0
	,10	1	,1	,1	11,1
	,12	6	,3	,3	11,4
	,13	4	,2	,2	11,7
	,15	1	,1	,1	11,7
	,17	7	,4	,4	12,1
	,17	264	14,2	14,9	27,0
	,18	1	,1	,1	27,0
	,20	1	,1	,1	27,1
	,23	2	,1	,1	27,2
	,25	266	14,3	15,0	42,2
	,25	8	,4	,5	42,6
	,27	4	,2	,2	42,9
	,28	5	,3	,3	43,2
	,33	162	8,7	9,1	52,3
	,37	3	,2	,2	52,5
	,42	13	,7	,7	53,2

KSQlatenciasono_FD_Decimal

		Frequency	Percent	Valid Percent	Cumulative Percent
	,50	355	19,1	20,0	73,2
	,58	3	,2	,2	73,4
	,62	1	,1	,1	73,4
	,67	26	1,4	1,5	74,9
	,75	57	3,1	3,2	78,1
	,83	3	,2	,2	78,3
	,83	6	,3	,3	78,6
	,87	2	,1	,1	78,7
	,92	2	,1	,1	78,8
	1,00	216	11,6	12,2	91,0
	1,08	1	,1	,1	91,0
	1,17	1	,1	,1	91,1
	1,25	2	,1	,1	91,2
	1,33	1	,1	,1	91,3
	1,50	33	1,8	1,9	93,1
	1,67	2	,1	,1	93,2
	2,00	86	4,6	4,8	98,1
	2,50	10	,5	,6	98,6
	3,00	20	1,1	1,1	99,8
	4,00	4	,2	,2	100,0
	Total	1775	95,5	100,0	
Missing	System	83	4,5		
Total		1858	100,0		

HorasEfectivasSono_WD_decimal

		Frequency	Percent	Valid Percent	Cumulative Percent
Valid	2,00	3	,2	,2	,2
	2,28	1	,1	,1	,2
	2,50	3	,2	,2	,4
	2,75	2	,1	,1	,5
	3,00	6	,3	,3	,9
	3,25	1	,1	,1	,9
	3,33	1	,1	,1	1,0
	3,50	8	,4	,5	1,4
	3,58	1	,1	,1	1,5
	3,72	1	,1	,1	1,5
	3,75	3	,2	,2	1,7
	3,83	1	,1	,1	1,8
	4,00	11	,6	,6	2,4
	4,08	1	,1	,1	2,4
	4,17	1	,1	,1	2,5
	4,17	1	,1	,1	2,6
	4,25	2	,1	,1	2,7
	4,33	1	,1	,1	2,7
	4,33	1	,1	,1	2,8
	4,42	2	,1	,1	2,9
	4,50	17	,9	1,0	3,9
	4,60	1	,1	,1	3,9
	4,67	2	,1	,1	4,0
	4,75	3	,2	,2	4,2
	4,83	4	,2	,2	4,4
	4,83	1	,1	,1	4,5
	5,00	32	1,7	1,8	6,3
	5,00	1	,1	,1	6,4
	5,08	1	,1	,1	6,4
	5,17	1	,1	,1	6,5
	5,17	2	,1	,1	6,6
	5,17	1	,1	,1	6,7
	5,20	1	,1	,1	6,7
	5,25	1	,1	,1	6,8
	5,25	13	,7	,7	7,5
	5,30	1	,1	,1	7,6
	5,33	1	,1	,1	7,6
	5,33	10	,5	,6	8,2
	5,33	1	,1	,1	8,2
	5,50	33	1,8	1,9	10,1
	5,58	1	,1	,1	10,2
	5,67	2	,1	,1	10,3

HorasEfectivasSono_WD_decimal

	Frequency	Percent	Valid Percent	Cumulative Percent
5,67	5	,3	,3	10,6
5,67	2	,1	,1	10,7
5,75	8	,4	,5	11,1
5,83	8	,4	,5	11,6
5,83	1	,1	,1	11,7
5,92	6	,3	,3	12,0
6,00	78	4,2	4,4	16,4
6,08	3	,2	,2	16,6
6,08	1	,1	,1	16,7
6,13	1	,1	,1	16,7
6,17	1	,1	,1	16,8
6,17	12	,6	,7	17,5
6,25	22	1,2	1,3	18,7
6,25	1	,1	,1	18,8
6,33	15	,8	,9	19,6
6,42	2	,1	,1	19,7
6,42	5	,3	,3	20,0
6,42	1	,1	,1	20,1
6,50	3	,2	,2	20,2
6,50	80	4,3	4,5	24,8
6,50	5	,3	,3	25,1
6,58	1	,1	,1	25,1
6,58	4	,2	,2	25,4
6,58	1	,1	,1	25,4
6,63	2	,1	,1	25,5
6,67	2	,1	,1	25,6
6,67	12	,6	,7	26,3
6,67	5	,3	,3	26,6
6,75	36	1,9	2,0	28,7
6,80	1	,1	,1	28,7
6,83	3	,2	,2	28,9
6,83	22	1,2	1,3	30,1
6,83	6	,3	,3	30,5
6,87	1	,1	,1	30,5
6,92	2	,1	,1	30,6
6,92	14	,8	,8	31,4
6,92	1	,1	,1	31,5
6,95	1	,1	,1	31,6
7,00	2	,1	,1	31,7
7,00	132	7,1	7,5	39,2
7,00	1	,1	,1	39,2
7,05	1	,1	,1	39,3

HorasEfectivasSono_WD_decimal

	Frequency	Percent	Valid Percent	Cumulative Percent
7,08	1	,1	,1	39,3
7,08	3	,2	,2	39,5
7,08	3	,2	,2	39,7
7,17	1	,1	,1	39,7
7,17	6	,3	,3	40,1
7,17	6	,3	,3	40,4
7,22	1	,1	,1	40,5
7,25	40	2,2	2,3	42,8
7,25	1	,1	,1	42,8
7,33	2	,1	,1	42,9
7,33	31	1,7	1,8	44,7
7,33	3	,2	,2	44,9
7,35	1	,1	,1	44,9
7,38	2	,1	,1	45,0
7,42	1	,1	,1	45,1
7,42	13	,7	,7	45,8
7,45	1	,1	,1	45,9
7,45	1	,1	,1	45,9
7,50	1	,1	,1	46,0
7,50	163	8,8	9,3	55,3
7,50	1	,1	,1	55,3
7,52	1	,1	,1	55,4
7,58	2	,1	,1	55,5
7,58	5	,3	,3	55,8
7,58	2	,1	,1	55,9
7,67	3	,2	,2	56,1
7,67	23	1,2	1,3	57,4
7,70	1	,1	,1	57,4
7,72	1	,1	,1	57,5
7,73	2	,1	,1	57,6
7,75	58	3,1	3,3	60,9
7,75	3	,2	,2	61,1
7,80	1	,1	,1	61,1
7,83	3	,2	,2	61,3
7,83	27	1,5	1,5	62,8
7,83	3	,2	,2	63,0
7,88	1	,1	,1	63,0
7,92	8	,4	,5	63,5
7,92	1	,1	,1	63,6
7,97	1	,1	,1	63,6
7,98	1	,1	,1	63,7
8,00	2	,1	,1	63,8

HorasEfectivasSono_WD_decimal

	Frequency	Percent	Valid Percent	Cumulative Percent
8,00	196	10,5	11,1	74,9
8,00	1	,1	,1	75,0
8,08	2	,1	,1	75,1
8,08	5	,3	,3	75,4
8,13	3	,2	,2	75,6
8,17	14	,8	,8	76,4
8,17	7	,4	,4	76,7
8,25	1	,1	,1	76,8
8,25	33	1,8	1,9	78,7
8,27	1	,1	,1	78,7
8,32	1	,1	,1	78,8
8,33	4	,2	,2	79,0
8,33	18	1,0	1,0	80,0
8,42	5	,3	,3	80,3
8,45	1	,1	,1	80,4
8,47	1	,1	,1	80,4
8,50	1	,1	,1	80,5
8,50	96	5,2	5,5	86,0
8,55	1	,1	,1	86,0
8,58	1	,1	,1	86,1
8,58	6	,3	,3	86,4
8,63	2	,1	,1	86,5
8,67	9	,5	,5	87,0
8,67	2	,1	,1	87,2
8,75	18	1,0	1,0	88,2
8,83	1	,1	,1	88,2
8,83	17	,9	1,0	89,2
8,85	1	,1	,1	89,3
8,92	7	,4	,4	89,7
8,92	1	,1	,1	89,7
8,97	1	,1	,1	89,8
9,00	72	3,9	4,1	93,9
9,08	2	,1	,1	94,0
9,17	2	,1	,1	94,1
9,25	7	,4	,4	94,5
9,33	1	,1	,1	94,5
9,33	6	,3	,3	94,9
9,42	7	,4	,4	95,3
9,50	21	1,1	1,2	96,5
9,58	1	,1	,1	96,5
9,63	1	,1	,1	96,6
9,67	3	,2	,2	96,8

HorasEfectivasSono_WD_decimal

		Frequency	Percent	Valid Percent	Cumulative Percent
	9,67	1	,1	,1	96,8
	9,75	2	,1	,1	96,9
	9,83	2	,1	,1	97,0
	10,00	18	1,0	1,0	98,1
	10,17	1	,1	,1	98,1
	10,25	4	,2	,2	98,4
	10,33	1	,1	,1	98,4
	10,50	9	,5	,5	98,9
	10,75	3	,2	,2	99,1
	10,83	4	,2	,2	99,3
	10,98	1	,1	,1	99,4
	11,00	3	,2	,2	99,5
	11,13	1	,1	,1	99,6
	11,17	1	,1	,1	99,7
	11,25	2	,1	,1	99,8
	11,33	1	,1	,1	99,8
	11,50	1	,1	,1	99,9
	11,50	1	,1	,1	99,9
	14,00	1	,1	,1	100,0
	Total	1759	94,7	100,0	
Missing	System	99	5,3		
Total		1858	100,0		

HorasEfectivasSono_FD_decimal

		Frequency	Percent	Valid Percent	Cumulative Percent
Valid	2,50	1	,1	,1	,1
	2,75	1	,1	,1	,1
	3,00	1	,1	,1	,2
	3,25	1	,1	,1	,2
	3,50	1	,1	,1	,3
	4,00	7	,4	,4	,7
	4,50	2	,1	,1	,8
	4,75	1	,1	,1	,9
	4,83	1	,1	,1	,9
	5,00	13	,7	,7	1,7
	5,33	1	,1	,1	1,7
	5,50	13	,7	,7	2,5
	5,75	2	,1	,1	2,6
	5,83	1	,1	,1	2,6
	5,92	1	,1	,1	2,7
	5,97	1	,1	,1	2,7
	6,00	39	2,1	2,2	5,0
	6,25	3	,2	,2	5,1
	6,33	1	,1	,1	5,2
	6,42	2	,1	,1	5,3
	6,50	41	2,2	2,3	7,7
	6,58	1	,1	,1	7,7
	6,65	1	,1	,1	7,8
	6,67	4	,2	,2	8,0
	6,75	9	,5	,5	8,5
	6,83	8	,4	,5	9,0
	6,83	2	,1	,1	9,1
	6,92	1	,1	,1	9,1
	7,00	83	4,5	4,7	13,9
	7,08	1	,1	,1	13,9
	7,17	1	,1	,1	14,0
	7,17	4	,2	,2	14,2
	7,25	13	,7	,7	15,0
	7,33	9	,5	,5	15,5
	7,33	1	,1	,1	15,5
	7,42	1	,1	,1	15,6
	7,50	90	4,8	5,1	20,7
	7,52	1	,1	,1	20,8
	7,58	1	,1	,1	20,9
	7,63	1	,1	,1	20,9
	7,63	1	,1	,1	21,0
	7,67	17	,9	1,0	21,9

HorasEfectivasSono_FD_decimal

	Frequency	Percent	Valid Percent	Cumulative Percent
7,67	1	,1	,1	22,0
7,72	2	,1	,1	22,1
7,75	1	,1	,1	22,2
7,75	31	1,7	1,8	23,9
7,82	1	,1	,1	24,0
7,83	1	,1	,1	24,1
7,83	23	1,2	1,3	25,4
7,83	1	,1	,1	25,4
7,87	1	,1	,1	25,5
7,92	13	,7	,7	26,2
7,97	2	,1	,1	26,3
8,00	209	11,2	11,9	38,3
8,08	2	,1	,1	38,4
8,15	1	,1	,1	38,5
8,17	10	,5	,6	39,0
8,25	15	,8	,9	39,9
8,33	23	1,2	1,3	41,2
8,42	7	,4	,4	41,6
8,50	134	7,2	7,7	49,3
8,58	1	,1	,1	49,3
8,58	1	,1	,1	49,4
8,63	1	,1	,1	49,4
8,67	16	,9	,9	50,3
8,67	2	,1	,1	50,5
8,75	34	1,8	1,9	52,4
8,83	1	,1	,1	52,5
8,83	34	1,8	1,9	54,4
8,85	1	,1	,1	54,5
8,92	11	,6	,6	55,1
8,95	1	,1	,1	55,1
8,97	1	,1	,1	55,2
9,00	195	10,5	11,1	66,3
9,08	2	,1	,1	66,5
9,13	1	,1	,1	66,5
9,17	9	,5	,5	67,0
9,17	1	,1	,1	67,1
9,22	1	,1	,1	67,1
9,23	1	,1	,1	67,2
9,25	36	1,9	2,1	69,3
9,33	15	,8	,9	70,1
9,42	8	,4	,5	70,6
9,42	1	,1	,1	70,6

HorasEfectivasSono_FD_decimal

	Frequency	Percent	Valid Percent	Cumulative Percent
9,48	1	,1	,1	70,7
9,50	101	5,4	5,8	76,5
9,58	3	,2	,2	76,6
9,67	19	1,0	1,1	77,7
9,73	1	,1	,1	77,8
9,75	31	1,7	1,8	79,5
9,77	1	,1	,1	79,6
9,83	23	1,2	1,3	80,9
9,92	16	,9	,9	81,8
9,95	1	,1	,1	81,9
10,00	92	5,0	5,3	87,1
10,10	1	,1	,1	87,2
10,13	1	,1	,1	87,3
10,17	5	,3	,3	87,5
10,20	1	,1	,1	87,6
10,25	8	,4	,5	88,1
10,33	1	,1	,1	88,1
10,33	13	,7	,7	88,9
10,42	3	,2	,2	89,0
10,47	1	,1	,1	89,1
10,48	1	,1	,1	89,1
10,50	48	2,6	2,7	91,9
10,58	1	,1	,1	91,9
10,67	8	,4	,5	92,4
10,73	1	,1	,1	92,5
10,75	12	,6	,7	93,1
10,83	12	,6	,7	93,8
10,88	3	,2	,2	94,0
10,92	5	,3	,3	94,3
10,98	1	,1	,1	94,3
11,00	39	2,1	2,2	96,6
11,17	1	,1	,1	96,6
11,25	5	,3	,3	96,9
11,33	4	,2	,2	97,1
11,42	1	,1	,1	97,2
11,50	9	,5	,5	97,7
11,67	2	,1	,1	97,8
11,75	6	,3	,3	98,2
11,83	1	,1	,1	98,2
11,92	2	,1	,1	98,3
12,00	10	,5	,6	98,9
12,33	2	,1	,1	99,0

HorasEfectivasSono_FD_decimal

		Frequency	Percent	Valid Percent	Cumulative Percent
	12,50	4	,2	,2	99,3
	12,67	3	,2	,2	99,4
	12,75	1	,1	,1	99,5
	12,83	1	,1	,1	99,5
	13,00	3	,2	,2	99,7
	13,50	1	,1	,1	99,8
	13,58	1	,1	,1	99,8
	14,00	1	,1	,1	99,9
	14,42	1	,1	,1	99,9
	14,50	1	,1	,1	100,0
	Total	1750	94,2	100,0	
Missing	System	108	5,8		
Total		1858	100,0		

KSQ_WeekAverageSleepDuration

		Frequency	Percent	Valid Percent	Cumulative Percent
Valid	,00	3	,2	,2	,2
	3,11	1	,1	,1	,2
	3,14	2	,1	,1	,3
	3,21	1	,1	,1	,4
	3,29	1	,1	,1	,5
	3,57	1	,1	,1	,5
	3,79	2	,1	,1	,6
	3,86	1	,1	,1	,7
	3,93	1	,1	,1	,7
	4,00	1	,1	,1	,8
	4,08	1	,1	,1	,9
	4,11	1	,1	,1	,9
	4,21	1	,1	,1	1,0
	4,24	1	,1	,1	1,0
	4,29	1	,1	,1	1,1
	4,32	1	,1	,1	1,1
	4,36	2	,1	,1	1,3
	4,38	1	,1	,1	1,3
	4,43	1	,1	,1	1,4
	4,50	2	,1	,1	1,5
	4,57	2	,1	,1	1,6
	4,63	1	,1	,1	1,7
	4,64	2	,1	,1	1,8
	4,68	1	,1	,1	1,8
	4,71	2	,1	,1	1,9

KSQ_WeekAverageSleepDuration

	Frequency	Percent	Valid Percent	Cumulative Percent
4,75	1	,1	,1	2,0
4,79	1	,1	,1	2,1
4,86	2	,1	,1	2,2
4,87	1	,1	,1	2,2
4,88	1	,1	,1	2,3
4,89	1	,1	,1	2,3
4,90	1	,1	,1	2,4
4,93	2	,1	,1	2,5
5,00	3	,2	,2	2,7
5,05	1	,1	,1	2,7
5,06	1	,1	,1	2,8
5,07	3	,2	,2	3,0
5,11	1	,1	,1	3,0
5,13	1	,1	,1	3,1
5,14	3	,2	,2	3,3
5,18	1	,1	,1	3,3
5,21	2	,1	,1	3,4
5,23	1	,1	,1	3,5
5,24	1	,1	,1	3,5
5,29	6	,3	,3	3,9
5,33	1	,1	,1	3,9
5,36	1	,1	,1	4,0
5,39	1	,1	,1	4,1
5,40	1	,1	,1	4,1
5,43	2	,1	,1	4,2
5,48	1	,1	,1	4,3
5,50	3	,2	,2	4,5
5,52	1	,1	,1	4,5
5,54	1	,1	,1	4,6
5,55	1	,1	,1	4,6
5,57	1	,1	,1	4,7
5,60	1	,1	,1	4,7
5,62	1	,1	,1	4,8
5,64	1	,1	,1	4,9
5,64	3	,2	,2	5,0
5,65	1	,1	,1	5,1
5,67	1	,1	,1	5,1
5,67	2	,1	,1	5,3
5,71	3	,2	,2	5,4
5,75	1	,1	,1	5,5
5,76	2	,1	,1	5,6
5,79	5	,3	,3	5,9

KSQ_WeekAverageSleepDuration

	Frequency	Percent	Valid Percent	Cumulative Percent
5,81	1	,1	,1	5,9
5,86	9	,5	,5	6,5
5,88	2	,1	,1	6,6
5,88	2	,1	,1	6,7
5,90	1	,1	,1	6,7
5,92	1	,1	,1	6,8
5,93	4	,2	,2	7,0
5,94	1	,1	,1	7,1
5,96	2	,1	,1	7,2
6,00	8	,4	,5	7,7
6,02	1	,1	,1	7,7
6,04	3	,2	,2	7,9
6,05	2	,1	,1	8,0
6,06	1	,1	,1	8,1
6,06	1	,1	,1	8,1
6,07	5	,3	,3	8,4
6,10	1	,1	,1	8,5
6,11	1	,1	,1	8,5
6,12	2	,1	,1	8,6
6,12	1	,1	,1	8,7
6,14	12	,6	,7	9,4
6,17	1	,1	,1	9,4
6,18	4	,2	,2	9,7
6,20	1	,1	,1	9,7
6,21	5	,3	,3	10,0
6,24	1	,1	,1	10,1
6,25	3	,2	,2	10,2
6,29	11	,6	,6	10,9
6,30	1	,1	,1	10,9
6,31	2	,1	,1	11,0
6,31	1	,1	,1	11,1
6,32	1	,1	,1	11,1
6,33	3	,2	,2	11,3
6,33	1	,1	,1	11,4
6,36	7	,4	,4	11,8
6,36	1	,1	,1	11,8
6,37	1	,1	,1	11,9
6,38	1	,1	,1	11,9
6,39	7	,4	,4	12,3
6,40	1	,1	,1	12,4
6,42	1	,1	,1	12,5
6,43	15	,8	,9	13,3

KSQ_WeekAverageSleepDuration

	Frequency	Percent	Valid Percent	Cumulative Percent
6,44	1	,1	,1	13,4
6,44	1	,1	,1	13,4
6,45	1	,1	,1	13,5
6,45	2	,1	,1	13,6
6,46	2	,1	,1	13,7
6,48	1	,1	,1	13,8
6,48	1	,1	,1	13,8
6,49	2	,1	,1	13,9
6,50	14	,8	,8	14,7
6,50	1	,1	,1	14,8
6,52	2	,1	,1	14,9
6,54	3	,2	,2	15,1
6,56	1	,1	,1	15,1
6,57	13	,7	,7	15,9
6,60	1	,1	,1	15,9
6,60	3	,2	,2	16,1
6,62	2	,1	,1	16,2
6,63	1	,1	,1	16,3
6,64	12	,6	,7	17,0
6,67	1	,1	,1	17,0
6,68	10	,5	,6	17,6
6,69	1	,1	,1	17,7
6,69	2	,1	,1	17,8
6,70	1	,1	,1	17,8
6,71	15	,8	,9	18,7
6,73	1	,1	,1	18,7
6,74	1	,1	,1	18,8
6,74	1	,1	,1	18,9
6,74	1	,1	,1	18,9
6,75	2	,1	,1	19,0
6,76	1	,1	,1	19,1
6,79	14	,8	,8	19,9
6,79	1	,1	,1	19,9
6,81	1	,1	,1	20,0
6,81	1	,1	,1	20,1
6,82	3	,2	,2	20,2
6,83	1	,1	,1	20,3
6,86	9	,5	,5	20,8
6,88	2	,1	,1	20,9
6,88	3	,2	,2	21,1
6,89	3	,2	,2	21,3
6,90	1	,1	,1	21,3

KSQ_WeekAverageSleepDuration

	Frequency	Percent	Valid Percent	Cumulative Percent
6,90	1	,1	,1	21,4
6,92	1	,1	,1	21,4
6,93	15	,8	,9	22,3
6,94	1	,1	,1	22,3
6,95	1	,1	,1	22,4
6,96	2	,1	,1	22,5
6,98	2	,1	,1	22,6
6,98	1	,1	,1	22,7
6,99	1	,1	,1	22,7
6,99	1	,1	,1	22,8
7,00	26	1,4	1,5	24,3
7,01	1	,1	,1	24,3
7,02	1	,1	,1	24,4
7,02	1	,1	,1	24,5
7,02	2	,1	,1	24,6
7,04	5	,3	,3	24,9
7,05	2	,1	,1	25,0
7,05	2	,1	,1	25,1
7,06	1	,1	,1	25,1
7,07	1	,1	,1	25,2
7,07	8	,4	,5	25,7
7,08	1	,1	,1	25,7
7,08	3	,2	,2	25,9
7,10	1	,1	,1	25,9
7,11	6	,3	,3	26,3
7,12	4	,2	,2	26,5
7,12	1	,1	,1	26,6
7,13	1	,1	,1	26,6
7,14	16	,9	,9	27,5
7,14	1	,1	,1	27,6
7,15	1	,1	,1	27,7
7,17	2	,1	,1	27,8
7,18	1	,1	,1	27,8
7,19	1	,1	,1	27,9
7,19	2	,1	,1	28,0
7,19	2	,1	,1	28,1
7,20	1	,1	,1	28,2
7,20	3	,2	,2	28,3
7,21	11	,6	,6	29,0
7,22	1	,1	,1	29,0
7,23	5	,3	,3	29,3
7,23	2	,1	,1	29,4

KSQ_WeekAverageSleepDuration

	Frequency	Percent	Valid Percent	Cumulative Percent
7,24	1	,1	,1	29,5
7,24	3	,2	,2	29,7
7,24	4	,2	,2	29,9
7,25	1	,1	,1	29,9
7,25	7	,4	,4	30,3
7,26	1	,1	,1	30,4
7,29	34	1,8	1,9	32,3
7,30	1	,1	,1	32,4
7,30	1	,1	,1	32,5
7,31	1	,1	,1	32,5
7,31	1	,1	,1	32,6
7,31	2	,1	,1	32,7
7,32	7	,4	,4	33,1
7,33	2	,1	,1	33,2
7,33	1	,1	,1	33,3
7,33	1	,1	,1	33,3
7,35	2	,1	,1	33,4
7,36	1	,1	,1	33,5
7,36	12	,6	,7	34,2
7,38	2	,1	,1	34,3
7,39	7	,4	,4	34,7
7,40	4	,2	,2	34,9
7,42	2	,1	,1	35,0
7,43	19	1,0	1,1	36,1
7,44	2	,1	,1	36,2
7,44	1	,1	,1	36,3
7,45	2	,1	,1	36,4
7,45	2	,1	,1	36,5
7,46	1	,1	,1	36,6
7,46	10	,5	,6	37,1
7,48	7	,4	,4	37,5
7,48	1	,1	,1	37,6
7,49	1	,1	,1	37,7
7,49	1	,1	,1	37,7
7,50	1	,1	,1	37,8
7,50	24	1,3	1,4	39,1
7,51	1	,1	,1	39,2
7,51	1	,1	,1	39,3
7,52	3	,2	,2	39,4
7,52	2	,1	,1	39,5
7,54	4	,2	,2	39,8
7,55	2	,1	,1	39,9

KSQ_WeekAverageSleepDuration

	Frequency	Percent	Valid Percent	Cumulative Percent
7,56	2	,1	,1	40,0
7,57	20	1,1	1,1	41,1
7,58	2	,1	,1	41,3
7,58	1	,1	,1	41,3
7,60	1	,1	,1	41,4
7,60	1	,1	,1	41,4
7,61	6	,3	,3	41,8
7,62	3	,2	,2	41,9
7,62	1	,1	,1	42,0
7,63	1	,1	,1	42,1
7,63	1	,1	,1	42,1
7,64	45	2,4	2,6	44,7
7,65	3	,2	,2	44,9
7,67	1	,1	,1	44,9
7,67	2	,1	,1	45,0
7,68	3	,2	,2	45,2
7,68	1	,1	,1	45,3
7,69	3	,2	,2	45,4
7,69	1	,1	,1	45,5
7,70	1	,1	,1	45,5
7,71	17	,9	1,0	46,5
7,71	1	,1	,1	46,6
7,72	1	,1	,1	46,6
7,73	1	,1	,1	46,7
7,73	1	,1	,1	46,7
7,73	2	,1	,1	46,9
7,74	2	,1	,1	47,0
7,75	9	,5	,5	47,5
7,75	1	,1	,1	47,5
7,76	6	,3	,3	47,9
7,76	6	,3	,3	48,2
7,77	5	,3	,3	48,5
7,79	1	,1	,1	48,6
7,79	24	1,3	1,4	49,9
7,80	1	,1	,1	50,0
7,81	1	,1	,1	50,1
7,81	1	,1	,1	50,1
7,81	2	,1	,1	50,2
7,81	1	,1	,1	50,3
7,82	11	,6	,6	50,9
7,82	1	,1	,1	51,0
7,83	1	,1	,1	51,0

KSQ_WeekAverageSleepDuration

	Frequency	Percent	Valid Percent	Cumulative Percent
7,83	7	,4	,4	51,4
7,85	4	,2	,2	51,7
7,86	22	1,2	1,3	52,9
7,87	1	,1	,1	53,0
7,88	1	,1	,1	53,0
7,88	1	,1	,1	53,1
7,89	4	,2	,2	53,3
7,90	3	,2	,2	53,5
7,90	1	,1	,1	53,5
7,92	1	,1	,1	53,6
7,92	3	,2	,2	53,8
7,93	35	1,9	2,0	55,8
7,94	1	,1	,1	55,8
7,94	1	,1	,1	55,9
7,94	1	,1	,1	55,9
7,96	10	,5	,6	56,5
7,97	1	,1	,1	56,6
7,98	6	,3	,3	56,9
7,98	2	,1	,1	57,0
7,99	3	,2	,2	57,2
8,00	1	,1	,1	57,3
8,00	47	2,5	2,7	59,9
8,00	1	,1	,1	60,0
8,01	1	,1	,1	60,1
8,02	1	,1	,1	60,1
8,02	5	,3	,3	60,4
8,04	11	,6	,6	61,0
8,05	4	,2	,2	61,3
8,07	16	,9	,9	62,2
8,07	1	,1	,1	62,2
8,08	1	,1	,1	62,3
8,08	1	,1	,1	62,3
8,10	2	,1	,1	62,5
8,10	3	,2	,2	62,6
8,11	10	,5	,6	63,2
8,12	3	,2	,2	63,4
8,13	1	,1	,1	63,4
8,14	30	1,6	1,7	65,1
8,14	1	,1	,1	65,2
8,17	5	,3	,3	65,5
8,17	1	,1	,1	65,5
8,18	7	,4	,4	65,9

KSQ_WeekAverageSleepDuration

	Frequency	Percent	Valid Percent	Cumulative Percent
8,19	3	,2	,2	66,1
8,19	4	,2	,2	66,3
8,20	1	,1	,1	66,4
8,20	1	,1	,1	66,5
8,21	14	,8	,8	67,3
8,21	1	,1	,1	67,3
8,23	2	,1	,1	67,4
8,23	1	,1	,1	67,5
8,24	1	,1	,1	67,5
8,24	4	,2	,2	67,8
8,25	1	,1	,1	67,8
8,25	1	,1	,1	67,9
8,26	2	,1	,1	68,0
8,27	2	,1	,1	68,1
8,29	48	2,6	2,7	70,9
8,31	1	,1	,1	70,9
8,31	2	,1	,1	71,0
8,32	6	,3	,3	71,4
8,33	1	,1	,1	71,4
8,33	4	,2	,2	71,7
8,35	2	,1	,1	71,8
8,36	20	1,1	1,1	72,9
8,36	1	,1	,1	73,0
8,38	1	,1	,1	73,0
8,39	4	,2	,2	73,3
8,40	3	,2	,2	73,4
8,40	1	,1	,1	73,5
8,41	1	,1	,1	73,5
8,42	1	,1	,1	73,6
8,43	20	1,1	1,1	74,7
8,44	1	,1	,1	74,8
8,44	1	,1	,1	74,9
8,45	1	,1	,1	74,9
8,46	8	,4	,5	75,4
8,48	3	,2	,2	75,5
8,48	1	,1	,1	75,6
8,49	2	,1	,1	75,7
8,50	22	1,2	1,3	77,0
8,51	1	,1	,1	77,0
8,51	1	,1	,1	77,1
8,52	1	,1	,1	77,1
8,52	3	,2	,2	77,3

KSQ_WeekAverageSleepDuration

	Frequency	Percent	Valid Percent	Cumulative Percent
8,54	2	,1	,1	77,4
8,55	3	,2	,2	77,6
8,55	1	,1	,1	77,7
8,56	2	,1	,1	77,8
8,56	2	,1	,1	77,9
8,56	1	,1	,1	77,9
8,57	21	1,1	1,2	79,1
8,57	1	,1	,1	79,2
8,58	1	,1	,1	79,3
8,60	4	,2	,2	79,5
8,60	1	,1	,1	79,5
8,60	1	,1	,1	79,6
8,61	5	,3	,3	79,9
8,62	1	,1	,1	79,9
8,62	1	,1	,1	80,0
8,63	2	,1	,1	80,1
8,64	20	1,1	1,1	81,3
8,65	1	,1	,1	81,3
8,67	2	,1	,1	81,4
8,68	6	,3	,3	81,8
8,68	1	,1	,1	81,8
8,69	2	,1	,1	81,9
8,70	1	,1	,1	82,0
8,71	18	1,0	1,0	83,0
8,71	1	,1	,1	83,1
8,72	1	,1	,1	83,1
8,73	2	,1	,1	83,3
8,73	1	,1	,1	83,3
8,74	2	,1	,1	83,4
8,75	3	,2	,2	83,6
8,77	1	,1	,1	83,7
8,77	1	,1	,1	83,7
8,79	17	,9	1,0	84,7
8,80	1	,1	,1	84,7
8,81	2	,1	,1	84,9
8,81	3	,2	,2	85,0
8,82	2	,1	,1	85,1
8,83	1	,1	,1	85,2
8,83	4	,2	,2	85,4
8,85	1	,1	,1	85,5
8,85	1	,1	,1	85,5
8,86	14	,8	,8	86,3

KSQ_WeekAverageSleepDuration

	Frequency	Percent	Valid Percent	Cumulative Percent
8,87	1	,1	,1	86,4
8,88	1	,1	,1	86,5
8,89	4	,2	,2	86,7
8,90	1	,1	,1	86,7
8,90	2	,1	,1	86,9
8,92	1	,1	,1	86,9
8,93	15	,8	,9	87,8
8,94	1	,1	,1	87,8
8,95	1	,1	,1	87,9
8,96	1	,1	,1	87,9
8,96	3	,2	,2	88,1
8,98	1	,1	,1	88,2
9,00	26	1,4	1,5	89,7
9,01	1	,1	,1	89,7
9,01	2	,1	,1	89,8
9,04	3	,2	,2	90,0
9,05	1	,1	,1	90,1
9,05	1	,1	,1	90,1
9,06	1	,1	,1	90,2
9,07	8	,4	,5	90,6
9,10	1	,1	,1	90,7
9,10	1	,1	,1	90,7
9,11	1	,1	,1	90,8
9,12	2	,1	,1	90,9
9,12	1	,1	,1	91,0
9,13	1	,1	,1	91,0
9,14	1	,1	,1	91,1
9,14	11	,6	,6	91,7
9,17	1	,1	,1	91,8
9,17	3	,2	,2	91,9
9,19	2	,1	,1	92,1
9,20	1	,1	,1	92,1
9,20	2	,1	,1	92,2
9,21	7	,4	,4	92,6
9,24	1	,1	,1	92,7
9,25	2	,1	,1	92,8
9,27	1	,1	,1	92,9
9,28	1	,1	,1	92,9
9,29	14	,8	,8	93,7
9,30	1	,1	,1	93,8
9,31	2	,1	,1	93,9
9,32	2	,1	,1	94,0

KSQ_WeekAverageSleepDuration

	Frequency	Percent	Valid Percent	Cumulative Percent
9,33	2	,1	,1	94,1
9,35	1	,1	,1	94,2
9,36	6	,3	,3	94,5
9,38	2	,1	,1	94,6
9,39	1	,1	,1	94,7
9,40	1	,1	,1	94,7
9,42	1	,1	,1	94,8
9,43	4	,2	,2	95,0
9,46	3	,2	,2	95,2
9,48	3	,2	,2	95,4
9,49	1	,1	,1	95,4
9,50	4	,2	,2	95,7
9,52	1	,1	,1	95,7
9,54	1	,1	,1	95,8
9,56	2	,1	,1	95,9
9,57	3	,2	,2	96,1
9,61	2	,1	,1	96,2
9,64	4	,2	,2	96,4
9,67	1	,1	,1	96,5
9,74	1	,1	,1	96,5
9,79	3	,2	,2	96,7
9,80	1	,1	,1	96,7
9,85	2	,1	,1	96,9
9,86	6	,3	,3	97,2
9,87	1	,1	,1	97,3
9,93	1	,1	,1	97,3
9,96	1	,1	,1	97,4
10,00	7	,4	,4	97,8
10,04	3	,2	,2	97,9
10,07	1	,1	,1	98,0
10,10	1	,1	,1	98,1
10,14	1	,1	,1	98,1
10,21	1	,1	,1	98,2
10,29	3	,2	,2	98,3
10,31	1	,1	,1	98,4
10,36	2	,1	,1	98,5
10,43	1	,1	,1	98,6
10,48	1	,1	,1	98,6
10,50	2	,1	,1	98,7
10,55	2	,1	,1	98,9
10,57	2	,1	,1	99,0
10,64	2	,1	,1	99,1

KSQ_WeekAverageSleepDuration

		Frequency	Percent	Valid Percent	Cumulative Percent
	10,71	1	,1	,1	99,1
	10,75	1	,1	,1	99,2
	10,79	1	,1	,1	99,3
	10,86	1	,1	,1	99,3
	10,93	1	,1	,1	99,4
	10,98	1	,1	,1	99,4
	10,98	1	,1	,1	99,5
	11,00	1	,1	,1	99,5
	11,04	1	,1	,1	99,6
	11,14	1	,1	,1	99,7
	11,17	1	,1	,1	99,7
	11,39	1	,1	,1	99,8
	11,60	1	,1	,1	99,8
	11,74	1	,1	,1	99,9
	11,76	1	,1	,1	99,9
	13,14	1	,1	,1	100,0
	Total	1750	94,2	100,0	
Missing	System	108	5,8		
Total		1858	100,0		

KSQqualidadesono

		Frequency	Percent	Valid Percent	Cumulative Percent
Valid	Muito bom	160	8,6	8,9	8,9
	Razoavelmente bom	713	38,4	39,8	48,7
	Nem bom nem mau	508	27,3	28,4	77,1
	Razoavelmente mau	340	18,3	19,0	96,1
	Muito mau	70	3,8	3,9	100,0
	Total	1791	96,4	100,0	
Missing	System	67	3,6		
Total		1858	100,0		
